# On the problems and promises of savanna fire regime change

**DOI:** 10.1038/s41467-021-25141-1

**Published:** 2021-08-12

**Authors:** Paul Laris

**Affiliations:** Geography Department, California State University, Long Beach, CA USA

**Keywords:** Biogeochemistry, Environmental sciences

**Arising from** Dr Geoffrey Lipsett-Moore et al. *Nature Communications* 10.1038/s41467-018-04687-7 (2021)

In their article, Lipsett-Moore et al.^1^ argue there exists a global opportunity for emissions reductions through early dry season (EDS) burning for 37 countries. They base their emissions reduction estimates on an approach developed in Australia and conclude that 20 least developed African countries account for 74% of the mitigation potential. While shifting fire regimes in savannas could possibly reduce emissions of GHGs, the approach used by the authors is overly simple and contains too many uncertainties for application in Africa. Indeed, it is probable that a shift to earlier burning will increase methane emissions there. Here we critically examine the problems with applying the model to Africa based on a literature review and our own field experiences and findings. We conclude with thoughts on alternatives.

There are four key issues with the proposed model. (i) There is an emissions trade-off when shifting fires earlier—although area burned declines, emission factors (EFs) increase due to combustion of uncured fuels. (ii) People in African savannas already set a large number of EDS fires; it is, therefore, questionable whether a shift to an earlier regime is feasible. (iii) The dichotomous model of early/late burning is flawed and the overly simplified reliance on the lowest rainfall month as a cut-off is unjustified. (iv) There remain problems with detecting and mapping patchy early fires. Finally, history is important here, for far too long non-indigenous experts have suggested, and often attempted to force changes to local burning practices. We find the proposed model ironic given that the EDS regime recommended is one that developed indigenously and has been applied by Africans for centuries.

## The emissions trade-off problem

GHG emissions from fires, such as methane, are determined by multiple factors, many of which change by season, but the proposed model only considers changes in the area burned. Critically, the model does not take into account the seasonal changes in EFs. In Africa, for example, EFCH_4_ increases by 50–400% when fires are earlier due to higher fuel moisture content^2–5^. As such, efforts to reduce emissions by burning earlier are misguided because the reduction in the burned area would be offset by the increase in EF resulting in higher methane emissions^4^. Indeed, authors of the Australian research, on which the model is based, have stated as much^6^.

## African early burning regimes

**Fig. 1:**
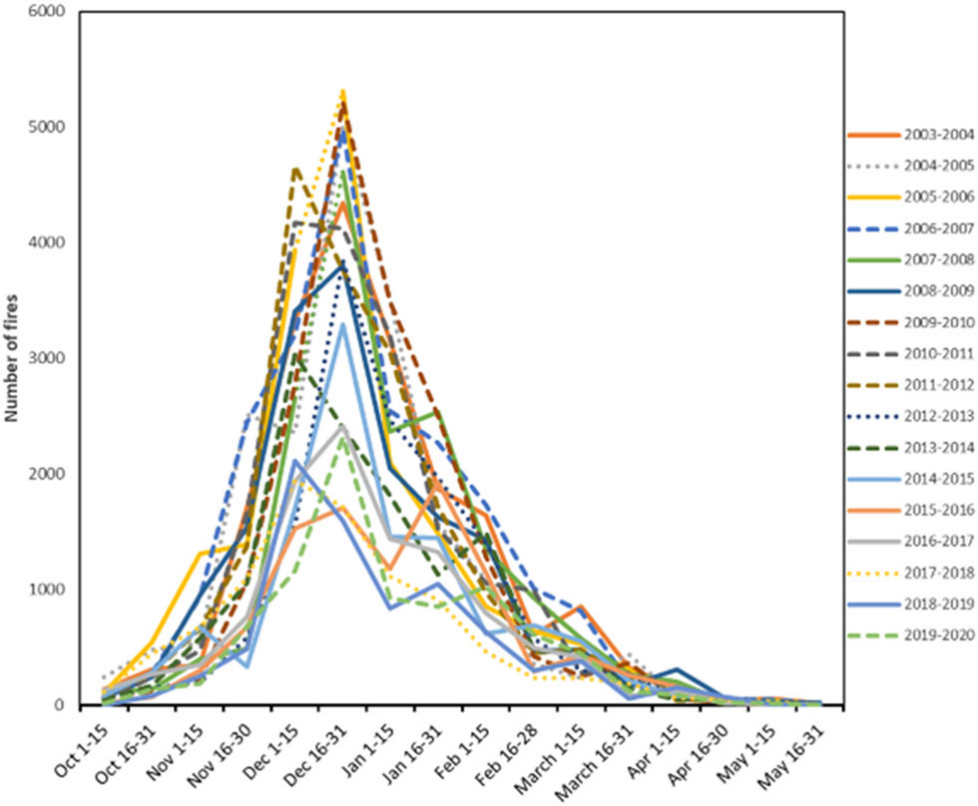
Number of active fires for an area of mesic savanna in West Africa covering portions of Mali, Guinea, Burkina Faso, and Ivory Coast with 750–1000 mm precipitation detected by MODIS over 15 years^9^. Note the high degree of regularity with a large number of fires lit early (prior to the government’s early fire cut-off of January 1). The recently documented decrease in fire is also apparent^24^.

In Africa, EDS burning is already widely practiced—the number and extent of fires typically peak prior to the middle of the dry season in the major savanna belts (see below)^7,8^. The temporal pattern of burning is very regular, indicating a systematic regime of EDS burning as shown in Fig. 1 for a sub-set of the mesic savanna belt in West Africa. This pattern reflects an intensive effort by thousands of people to set fires early and systematically^7–9^. The regular annual pattern is a function of people selecting and burning specific patches of vegetation that are just dry enough to burn^10,11^. These regimes of burning create patch-mosaics of previously burned, newly burned, and unburned vegetation—a process which reflects how the well-timed fires burn only grasses that have sufficiently cured, leaving the adjacent uncured grasses unburned^8,10^. As such, we question that additional vegetation could be burned early and wonder who would do the burning, and what would be the emissions impact of burning more uncured grasses? Importantly, increasing EDS burned area may not be possible due to both ecological (e.g., prevalence of too moist perennial grasses) and socio-economic factors, including constraints posed by agriculture. Increasing the amount of area burned early would require more fires being set during the harvest season and would undoubtedly result in crop damage^12^.

## The problematic early/late fire dichotomy

At the core of the proposed model is the application of an early/late fire dichotomy, which itself derives from a series of colonial-era experiments conducted in Africa and later repeated globally. These works examined the impacts of fires set near the beginning and end of the fire season, not the middle, which is when most fires occur^13^. Emissions from fires are a function of a suite of variables that are associated with fuel conditions and weather, which determine the key fire characteristics. These vary by season (as well as spatially) and by fire type. Wind, to give just one example, is critical and peaks in the middle season in parts of Africa. Research also finds that fire type (head or back) has a significant impact on fire intensity, combustion efficiency, and methane EF, yet no mention of fire type appears in the policy^14^.

**Fig. 2:**
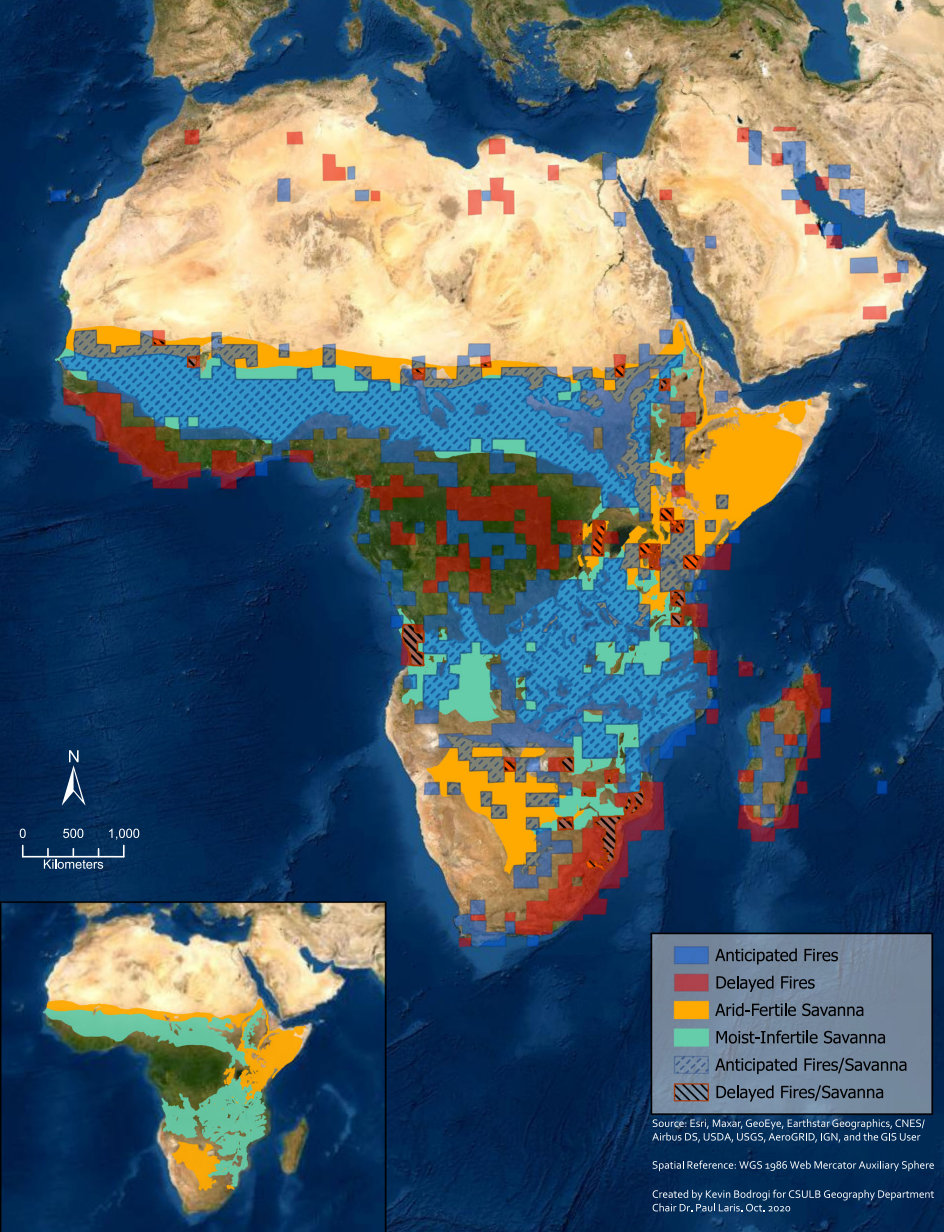
Map of early (anticipated) and late (delayed) fire seasons for Africa modified from Le page et al.^7^ over White’s savanna vegetation map^25^. Using threshold at ±30 days of difference between the values per grid cell for the middle of the fire season and middle of the CBI season, less than 6% of African savannas burn late. (Sources: Source: Esri, Maxar, GeoEye, Earthstar Geographics, CNES/ Airbus DS, USDA, USGS, AeroGRID, IGN, and the GIS User Community. Created by Kevin Bodrogi).

Critically, the authors offer no justification for their crude method of using months of least rainfall to distinguish early from late burning. There are far better alternatives such as vegetation dryness indexes^15^ or the Chandler Burning Index (CBI) both of which have been measured remotely and which provide a good indication of fuel moisture level at a scale reasonable for determining potential fire conditions. Indeed, Le Page et al. analyzed the timing of fire regimes globally compared to peak biomass dryness using CBI. They found that most African savannas burn at least 30 days prior to peak dryness (Fig. 2)^7^. This figure is in stark contrast with that used by Lipsett-Moore et al., which shows many savanna countries with predominantly LDS fire regimes. It is also noteworthy that Le Page used a grid-cell basis for their analysis that more accurately estimates the conditions of the fuels, compared with the crude country-level estimates based on rainfall used by Lipsett-Moore et al.

## Missing the early fires problem

It is well-documented that mapping of burned areas produced by a regime of patchy fires contains high levels of uncertainty; what is less well-known is that most patch-mosaic burning begins early. Although algorithms to estimate burned areas have improved, the commonly used ones tend to underestimate smaller fires resulting in significant underestimates of burned area and emissions from fires in Africa^16^. Critically, the fires missed are most often early, patchy ones^17^ posing two problems for the model. First, existing maps likely underestimate the amount of early burning making the use of a simple EDS/LDS ratio biased. And second, it would be difficult to monitor any shift in burning resulting from a policy change.

## Historical matters

The proposed policy for a regime of early burning is hardly new but has been practiced in Africa before. French and British colonial foresters also considered early fire preferable to late and when they did, colonial powers promoted a burning regime that was already widely practiced by the indigenous population. Our research documented how French foresters, realizing the impossibility of fire suppression, shifted their policy to promote early fires—a practice developed and used first by Africans^18^.

## Conclusions

Although well-intended, the model to reduce GHG emissions by selecting countries based on their presumed high LDS fire is flawed and unlikely to succeed. Indeed, if the numbers used by the IPCC are correct, a shift to earlier fires would cause an increase in methane emissions due to increased burning of uncured fuels. Rather than rework the proposed model, we suggest a better approach begins with the wealth of existing studies that seek solutions combining local practice with forestry department objectives^19,20^. In addition, it is critical to remember, that the existing burning regimes of Africans serve a plethora of purposes including reducing fire hazards and supporting biodiversity^10^. The types of fires which preserve human livelihoods and biodiversity are not always aligned with the goal of reducing GHG concentrations^8^. As such, changes in burning may result in unforeseen negative consequences^21^. Finally, recent research from Australia, where the proposed model originates, cautions against overly simple solutions to the fire emissions problem^22^.

We agree there is a need to modify fire regimes to reduce LDS fires in some places. Indeed, we often find this to be a goal of local populations. In cases we have studied, changing fire is not a matter of simply increasing early fire, but a complex management goal requiring strategies to maximize the good aspects of fire while minimizing the bad ones in accordance with local desires. A better approach would be to begin by studying how African people use strategic burning to render the landscape useful; only then can we begin to work with people to understand their needs and jointly develop methods to prevent unwanted fires. This will require collaborative efforts, community organizing, and smart technologies such as drones for mapping and smartphones for improving communication.

We end by noting the irony of the proposed solution of an EDS fire policy for the Australian context. This is, in effect, an effort to reintroduce the fire regime that was lost when the Aboriginal burning practices were suppressed and people were removed from the landscape^23^. In Africa, where the practice of EDS fires has largely persisted—in spite of suppression efforts—we suggest compensating the thousands of people for the ecosystem services they provide by setting fires early.

## Data Availability

This commentary does not include original data. All data referred to in the text have been published elsewhere. Data are accessible through the project website at http://www.cla.csulb.edu/departments/geography/savannalabo/data/ under the heading, “Mali Fire”.

## References

[1] Lipsett-Moore G (2018). Emissions mitigation opportunities for savanna countries from early dry season fire management. Nat. Commun..

[2] Intergovernmental Panel on Climate Change (IPCC). Good Practice Guidance and Uncertainty Management in National Greenhouse Gas Inventories, IPCC National Greenhouse Gas Inventories Programme, Hayama, Kanagawa, Japan (2000).

[3] Hoffa EA (1999). Seasonality of carbon emissions from biomass burning in a Zambian savanna. J. Geophys. Res..

[4] Korontzi S, Justice CO, Scholes RJ (2003). Influence of timing and spatial extent of savanna fires in southern Africa on atmospheric emissions. J. Arid Environ..

[5] Korontzi S (2005). Seasonal patterns in biomass burning emissions from southern African vegetation fires for the year 2000. Glob. Change Biol..

[6] Meyer CP (2012). Direct measurements of the seasonality of emission factors from savanna fires in northern Australia. J. Geophys. Res..

[7] Le Page Y, Duarte O, Jonsson P, Pereira J (2010). Seasonality of vegetation fires as modified by human action: observing the deviation from eco-climatic fire regimes. Glob. Ecol. Biogeogr..

[8] Archibald S (2016). Managing the human component of fire regimes: lessons from Africa. Philos. Trans. R. Soc. B.

[9] Laris P, Dadashi S, Jo A, Wechsler S (2016). Buffering the savanna: fire regimes and disequilibrium ecology in West Africa. Plant Ecol..

[10] Laris P (2002). Burning the seasonal mosaic: preventative burning strategies in the Wooded Savanna of Southern Mali. Hum. Ecol..

[11] Laris P (2011). Humanizing savanna biogeography: linking human practices with ecological patterns in a frequently burned savanna of southern Mali. Ann. Assoc. Am. Geogr..

[12] Laris P (2013). Integrating land change science and savanna fire models in West. Afr. Land.

[13] Laris P, Kone M, Dembele F (2017). The early/late fire dichotomy: time for a reassessment of Aubréville’s Savanna fire experiments. Prog. Phys. Geogr..

[14] Keene WC (2006). Emissions of major gaseous and particulate species during experimental burns of southern African biomass. J. Geophys. Res..

[15] Rahimzadeh-Bajgiran P (2012). Comparative evaluation of the Vegetation Dryness Index (VDI), the Temperature Vegetation Dryness Index (TVDI) and the improved TVDI (iTVDI) for water stress detection in semi-arid regions of Iran. ISPRS J. Photogramm. Remote Sens..

[16] Ramo R (2021). African burned area and fire carbon emissions are strongly impacted by small fires undetected by coarse resolution satellite data. Proc. Natl Acad. Sci. USA.

[17] Laris P (2005). Spatiotemporal problems with detecting seasonal mosaic fire regimes with coarse-resolution satellite data in savannas. Remote Sens. Environ..

[18] Laris P, Wardell DA (2006). Good, bad or “necessary evil?”: reinterpreting the colonial burning experiments in the savanna landscapes of West Africa. Geogr. J..

[19] Eloy L (2018). From fire suppression to fire management: advances and resistances to changes in fire policy in the savannas of Brazil and Venezuela. Geogr. J..

[20] Bilbao BA (2010). Indigenous use of fire and forest loss in Canaima National Park, Venezuela. Assessment of and tools for alternative strategies of fire management in pemón indigenous. Landsc. Ecol..

[21] Corey, B. et al. Better biodiversity accounting is needed to prevent bioperversity and maximize co‐benefits from savanna burning. *Conserv. Lett.* **13**, e12685, 10.1111/conl.12685 (2020).

[22] Murphy BP, Prior LD, Cochrane MA, Williamson GJ, Bowman DM (2019). Biomass consumption by surface fires across Earth’s most fire prone continent. Glob. Change Biol..

[23] Russell-Smith J (2003). Contemporary fire regimes of northern Australia, 1997–2001: change since Aboriginal occupancy, challenges for sustainable management. Int. J. Wildland Fire.

[24] Andela N (2017). A human-driven decline in global burned area. Science.

[25] White F (1983). The Vegetation of Africa.

